# Trends in Opioid Prescribing Following Pennsylvania Statewide Implementation of a Prescription Drug Monitoring Program

**DOI:** 10.7759/cureus.27879

**Published:** 2022-08-11

**Authors:** Chaim Miller, Asif M Ilyas

**Affiliations:** 1 Rothman Opioid Foundation, Rothman Orthopaedic Institute at Thomas Jefferson University, Philadelphia, USA; 2 Orthopaedic Surgery, Sidney Kimmel Medical College, Philadelphia, USA

**Keywords:** trends, legislation, pennsylvania, prescription drug monitoring program, drug overdose, opioid abuse, opioids

## Abstract

Background: The opioid epidemic is a major public health crisis in the United States. Legislators have enacted various strategies to combat this crisis, including the implementation of statewide prescription drug monitoring programs (PDMP). These PDMPs are electronic databases that collect and analyze patient prescription data on controlled substances, allowing physicians to review prior prescriptions before prescribing. The objective of this study was to determine opioid prescribing patterns after the implementation of a statewide PDMP in Pennsylvania.

Methods: After IRB approval, PDMP data were obtained from the Pennsylvania Department of Health. Data obtained included: drug name, days supplied, refill count, and partially filled prescriptions. The study timeline was three years, from first quarter 2017 through first quarter 2020.

Results: Over the three years post-implementation of a PDMP, Pennsylvania saw a 33% decrease in the overall quantity of opioid pills prescribed (677,194 absolute reduction), a 9% decrease in partially filled prescriptions (5,821 absolute reduction), and an 18% decrease in authorized refills (525 absolute reduction). Opioid prescriptions for greater than seven days of supply decreased by a larger amount than prescriptions for less than seven days of supply (43% vs 27%). Similarly, prescriptions for more than 22 pills saw a greater decrease than prescriptions for less than 21 days (37% vs 21%). However, the rate of decrease in opioid pills prescribed lessened from 14% in the first two years post implementation, to 10% in the third year. The decrease in partially filled opioid prescriptions for the first two years averaged 14% per year, while it increased by 23% in the third year. An 8% average decrease occurred in the rate of refills for opioid prescriptions for the first two years post implementation, followed by a 3% reduction in the third year.

Conclusion: There was a 33% decrease in the overall quantity of opioid pills prescribed in the three years after the implementation of the PDMP. The first two years after implementation saw the largest decreases in prescribing habits, which slowed in the third year. More data are needed to show the long-term effects of implementing a statewide PDMP.

## Introduction

From 1999 to 2017, almost 218,000 people died from opioid-related overdoses, with more than 11 million people having misused prescription opioids in 2017 alone [1,2]. Data from 2018 showed that approximately 128 people in the U.S. die every day from an opioid overdose [3]. A significant effort has been made to try and address this growing concern, which has led to the implementation of multiple programs aimed at combating different aspects of the problem. Among the 38 states with available prescription opioid-related overdose death data, 17 states saw a decline between 2017 and 2018 and none experienced a significant increase [4].

A prominent way states are tackling this crisis is by implementing a state-wide prescription drug monitoring program (PDMP). State PDMPs are electronic databases that collect and analyze patient prescription data on controlled substances and allow physicians the ability to access that information before prescribing. To date, all 50 states have implemented or are in the process of implementing a PDMP. However, the existing literature is mixed on the overall success of the PDMP. Several early studies have shown no effect on opioid prescribing, while some have found significant decreases in schedule ll opioids following the implementation of a PDMP [5,6].

Pennsylvania’s PDMP was established in August of 2016 and was mandated for use in specific situations on January 1, 2017 [7]. Being a new PDMP, little research has been done on the effectiveness of this program and how it compares to other states. The aim of this study is to contribute to the growing knowledge about statewide PDMPs. Specifically, this study aims to outline the three-year change in prescribing habits alongside the implementation of Pennsylvania’s PDMP.

This article was first posted on March 15, 2021, to the Research Square preprint server.

## Materials and methods

Data pertaining to opioid prescribing habits as listed below was obtained from the Pennsylvania Department of Health (PA DOH), which administers the PDMP in Pennsylvania. A request for data was filed and approved by the PA DOH along with IRB approval for this project (Thomas Jefferson University Institutional Review Board, Control # 20E.1221). The data were delivered and were recent to November 24, 2020. The PDMP monitors schedule II through schedule V controlled substances. As of January 1, 2017, all prescribers who are licensed, registered, or otherwise lawfully authorized to distribute, dispense, or administer a controlled substance, another drug, or device in the course of professional practice or research in the Commonwealth are required to register with and query the PDMP in certain situations.

Data obtained from the PDMP database include days supplied, quantity supplied, partial fills, and authorized refill count. Quantity was defined as the total number of opioid pills prescribed. Partial fill was defined as the number of prescriptions for opioids that were only partially filled. The refill count was defined as the total number of refills prescribed for opioid medication. In order to protect patient confidentiality, the PA DOH notes that values between 1 and 5 have been suppressed in the data reporting. If the value from only one group (e.g., county) during any given quarter required suppression, the next lowest value has also been suppressed. Authorized refill count data reflects the number of prescriptions with an authorized refill.

Data provided by the PA DOH are categorized by quarters, with each equaling three months of the year. Since the PA PDMP was implemented in late 2016, the fourth quarter of 2016 was ignored, and the study analyses began from Q1 2017 through Q1 2020.

## Results

**Figure 1:**
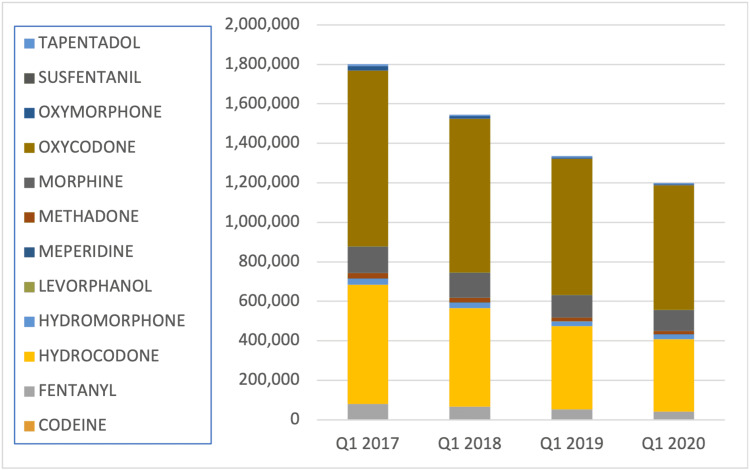
Opioid quantity of pills prescribed between Q1 2017 until Q1 2020. Pennsylvania’s PDMP was required for use in specific instances on January 1, 2017.

There was a 33% (677,194 absolute) decrease in the overall quantity of opioids prescribed from Q1 2017 through Q1 2020 (Figure 1). The largest absolute decrease in prescriptions was from Oxycodone (258,727 or 29%) and Hydrocodone (236,868 or 39%). During those three years, there was a 9% decrease (5,821 absolute) in partially filled prescriptions and an 18% decrease (525 absolute) in the authorized refill count.

**Figure 2:**
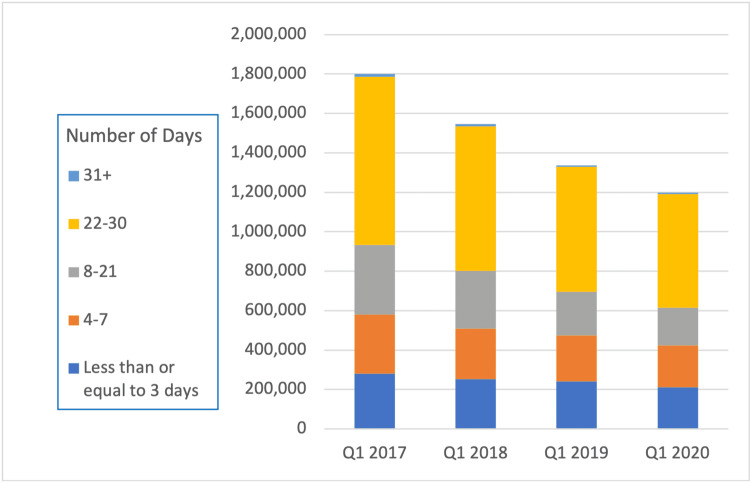
Days supplied for opioid prescriptions between Q1 2017 until Q1 2020.

**Figure 3:**
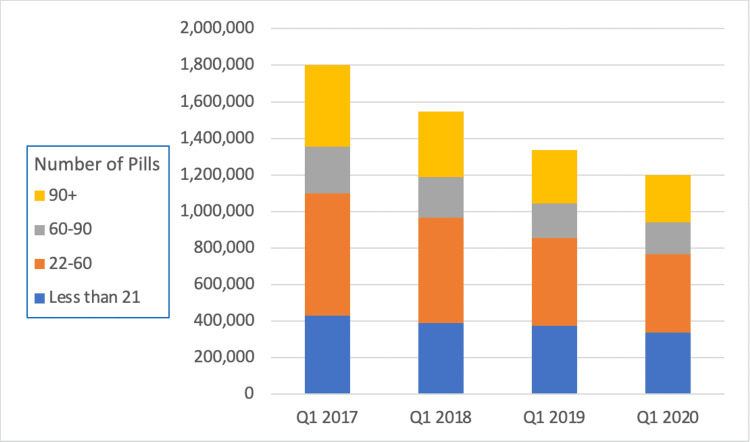
Opioid pill quantity prescribed between Q1 2017 until Q1 2020.

Opioid prescriptions for more than seven days of supply decreased by a larger amount than prescriptions for less than seven days of supply (43% vs 27%). Similarly, prescriptions for more than 22 pills saw a greater decrease than prescriptions for less than 21 days (37% vs 21%, Figures 2, 3).

**Figure 4:**
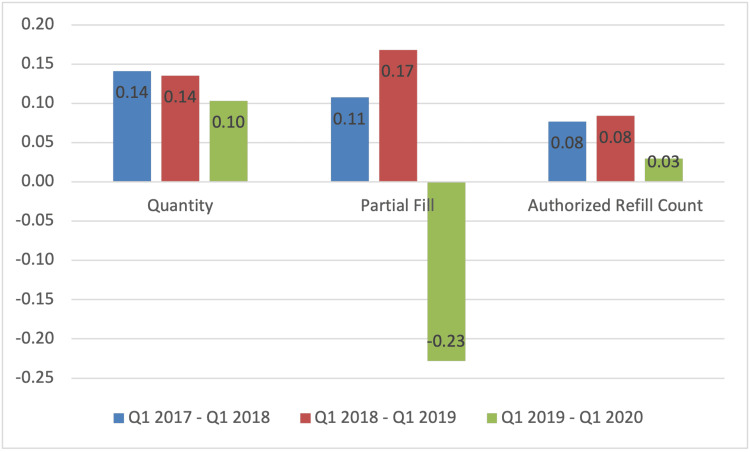
Rate of decrease for opioid prescribing habits between Q1 2017 until Q1 2020.

However, the rate of decrease in opioid pills prescribed per year decreased from 14% in the first two years after PDMP implementation to 10% in the third year. Partially filled opioid prescriptions decreased by an average of 14% per year for the first two years, while they increased by 23% in the third year. Finally, an 8% average decrease occurred in the rate of refills for opioid prescriptions for the first two years post-implementation, followed by a 3% in the third year (Figure 4).

## Discussion

Since the implementation of a new statewide PDMP, there has been a 33% decrease in the overall quantity of opioids prescribed, a 9% decrease in partially filled prescriptions, and an 18% decrease in the authorized refill count in the state of Pennsylvania. We found a larger decrease in long-term prescriptions of opioids compared to short-term prescriptions, as well as decreasing rates of decline in the third-year post-implementation.

Perhaps the most clinically significant aspect of the data presented above is the larger decrease in longer-term opioid prescriptions compared with short-term prescriptions. Currently, as many as one in four patients receiving long-term opioid therapy in a primary care setting struggles with opioid addiction. The longer a patient is on opioids, the higher the likelihood of dependence on the medication and, thereby, the higher the potential for abuse [8,9].

Multiple nearby states and areas have published similar opioid reductions alongside a statewide PDMP. In New York, the implementation of a PDMP (I-STOP law) led to a significant decrease in rates of potentially problematic patterns of opioid prescribing [10], as well as a leveling off of prescription opioid mortality [11]. In Pittsburgh, Pennsylvania, a PDMP use mandate was associated with fewer patients prescribed opioids in the emergency department than pre-PDMP implementation [12]. One study found a 30% reduction in the self-reported rate of schedule ll prescriptions among patients reporting pain as a reason for a visit. Data from the state of Florida demonstrated that after the implementation of the PDMP, oxycodone-caused mortality abruptly declined by 25% [13], opioid prescriptions declined by 1.4%, opioid volume decreased by 2.5%, and MME per transaction decreased by 5.6% [14].

Since PDMPs are the most researched program established to combat the opioid crisis, there exist multiple studies that investigate the various aspects of what makes an effective PDMP. Since PDMPs vary by state, it would make sense that certain states will see significant effectiveness and others would not. Multiple studies have shown decreases in opioid-related overdoses and deaths, especially the more "robust" a PDMP is [15-20]. "Robustness" with regards to a PDMP may include facets such as use and registration mandate, delegate access, proactive reporting, no prescriber immunity for failing to query the PDMP, as well as reporting data to multiple neighboring states [21]. Currently, Pennsylvania’s PDMP includes registration and comprehensive use mandate, proactive reporting, and delegate access, but does provide prescriber immunity for failing to query the PDMP [22]. This enhances our understanding that effective PDMPs need to be comprehensive and robust to have a significant effect in combating the opioid crisis.

The implementation of statewide PDMPs has brought along some significant challenges for prescribers and others, both intentionally and unintentionally. For example, three studies have found an association between PDMP implementation and heroin overdose death rates [23,24]. This is hypothesized to be due to the intended consequences of lowering prescription opioids in the general public [25]. This is mirrored in a qualitative study done in Philadelphia and San Francisco, which documented the transition from prescription opioids to heroin. They found that most heroin users were originally prescribed opioids but found heroin to be a more available and inexpensive option when the supply of opioids became too small and too costly [26]. Heroin-related overdose deaths in Pennsylvania have remained stagnant since 2016 [27]. Cases like this show the importance that practitioners have in referring and encouraging those at risk for opioid abuse to seek treatment at treatment centers or by other evidence-based treatment methods.

Healthcare providers have also outlined the problems with implementing and integrating a statewide PDMP-especially a mandatory one-on workflow and time-sensitive situations [28]. Any online database that is required to be queried in specific situations can lead to uncertainty if the program is not working properly and does not adequately address what prescribers should do when presented with the opioid prescribing history of their patients. If policymakers wish to effectively implement a PDMP, it is vital that the program be integrated smoothly into daily workflow and have a contingency plan in case the program is not accessible. Also needed is support for including trend data and enhanced patient profiles that include additional data beyond controlled substances prescribed. This process could be optimized by including frequent and effective collaboration between all stakeholders involved [29].

Pennsylvania’s PDMP was the first program to effectively track opioids prescribed and filled within the state. Due to incomplete data prior to its implementation, a major limitation is that this paper looked at opioid trends following - and not directly prior to - its implementation. It is necessary to note that while the trends of opioids prescribed showed a decrease, this may not be purely related to the PDMP. Increased education, the PDMP use and registration mandate, and the mandate for electronic prescribing of controlled substances, among other public health measures, could have also influenced the observed trend.

## Conclusions

Currently, opioid prescribing data are only available for three years post-implementation in Pennsylvania. Our study found a decline in opioid prescribing habits, including the number of opioids prescribed, the number of days prescribed, and opioid prescription refills. While a significant decrease has been shown, monitoring for external and unintended consequences of decreased opioid prescribing is important. Statewide PDMPs have been the focus of targeted opioid legislation in recent years. Thoughtful consideration is paramount to the successful integration of the PDMP into the physician workflow. Alongside implementation, targeted training for healthcare providers is necessary and could be the differentiation factor for a successful PDMP. PDMP results are promising, but long-term studies are needed to address its lasting impact on opioid prescribing habits.
